# Understanding Australian Cat Caregiver Motivations and Reactions to Behaviour-Change Messaging on Cat Containment: Insights for Campaign Design

**DOI:** 10.3390/ani16050784

**Published:** 2026-03-03

**Authors:** Gemma C. Ma, Kiara L. Speedy, Patricia David, M. Carolyn Gates, Katherine E. Littlewood, Sarah Zito

**Affiliations:** 1 The Royal Society for the Prevention of Cruelty to Animals New South Wales, Yagoona, NSW 2199, Australia; 2 Sydney School of Veterinary Science, The University of Sydney, Camperdown, NSW 2006, Australia; 3 Khemistry, Brisbane, QLD 4000, Australia; 4 AkoVet Limited, Palmerston North 4410, New Zealand; 5 RSPCA Australia, Deakin West, ACT 2600, Australia

**Keywords:** behaviour change, cat containment, motivation, marketing campaign, feline welfare

## Abstract

Domestic cat containment has become an increasing focus of companion animal management in Australia, yet uptake among caregivers who allow their cats to roam remains limited. This study explored how caregivers with established outdoor routines but no strong opposition to containment perceive their cats, interpret risk, and respond to different campaign framings. Four online focus groups were conducted in New South Wales with 22 cat caregivers who allowed their cats outdoor access. Thematic analysis indicated that containment decisions, as interpreted through the analytic process, were shaped by how cats were acquired, prior outdoor experience, beliefs about how cats exercise agency, and beliefs about what factors contribute to feline welfare. Caregivers frequently minimised risk and believed their cats could manage themselves outdoors, reinforcing resistance to change. Reactions to the two tested campaign concepts indicated that messages perceived as moralising or fear-based elicited guilt and avoidance. In contrast, caregivers preferred solution-focused messages showing how cats could thrive safely at home. The findings suggest that once roaming behaviours are established, emotional and habitual barriers make voluntary change difficult. Effective interventions should combine motivational framing with clear, achievable actions and target owners at early decision points, particularly adoption, when new habits and expectations about cat management are more readily established.

## Simple Summary

Keeping pet cats safely at home is increasingly promoted in Australia to protect cats, wildlife, and communities, yet many cat caregivers are reluctant to change established outdoor routines. This study explored how people who allow their cats to roam think about their cats’ needs, safety, and overall wellbeing, and how they respond to different public messages about keeping cats at home. We held online group discussions with 22 cat caregivers in New South Wales who currently allow outdoor access but were not strongly opposed to change. Participants often believed their cats could look after themselves outdoors and downplayed risks such as injury, disease, or getting lost. These beliefs were shaped by how the cat was acquired, how long it had roamed outdoors, and ideas about cats’ independence and happiness. Messages that focused on blame, fear, or moral judgment made people feel guilty or defensive and were usually rejected. In contrast, practical and positive messages showing how cats can live happy, active, and safe lives at home were better received. The findings suggest that changing roaming habits is hardest once routines are established. Efforts to encourage safer cat management are likely to be more effective if they focus on support, clear solutions, and early decision points such as adoption.

## 1. Introduction

The domestic cat (*Felis catus*) remains one of Australia’s most popular companion animals, with ownership rates rising from 27% of households in 2019 to 34% in 2025, equating to an estimated 5.8 million owned cats nationwide [1]. While cats are valued companions to many households, their free-roaming behaviour has been implicated in negative impacts on native wildlife, ecosystems, and communities, particularly in areas where species have not evolved alongside feline predators [2]. As such, there has been growing interest in promoting cat containment as a practical strategy to reduce predation and nuisance behaviours [3], while also lowering the risks of injury, poisoning, and disease transmission for the cats themselves [4,5]. Containment encompasses a spectrum of approaches, from dusk-to-dawn curfews through to 24 h confinement to the owner’s property using cat-proof fencing, enclosures, or indoor-only housing [6], boundary deterrent devices [7], or supervised outdoor access such as leash-walking [8]. Some jurisdictions with sensitive habitats have also introduced cat exclusion zones to eliminate cat presence entirely; however, these are regulatory access restrictions rather than owner-implemented containment measures [9].

A national representative survey of pet ownership in Australia showed that the proportion of Australian cat owners keeping their cats strictly indoors has increased steadily from 36% in 2019 to 42% in 2022 and 48% in 2025 [1]. This trend might reflect the introduction of stricter cat curfews in some states as well as growing public concern about the risks associated with outdoor access [10]. Despite this trend, around half of owners continue to allow their cats outdoors, and among these, most (74%) permit unrestricted roaming, with only a minority (26%) using leads or cat runs [1]. These figures indicate that a substantial proportion of cat caregivers would still need to change their behaviour for universal containment to be achieved. At the same time, there is evidence that the transition to indoor lifestyles is not always managed in ways that consider feline welfare, with some cats exhibiting behavioural indicators consistent with negative affective experiences [11]. An online survey of over 12,000 Australian cat caregivers demonstrated that many Australian cats lack sufficient opportunities for stimulation and exercise [12], which increases the risk of frustration, anxiety, inactivity, and undesirable behaviours [13,14,15]. Such welfare considerations highlight the importance of ensuring that containment strategies not only are effective for biodiversity protection but also promote positive feline welfare. Together, these patterns underscore the need for well-designed behaviour-change initiatives that both motivate and support owners in adopting safe, sustainable, and welfare-enhancing containment practices.

Understanding how to motivate and support owners to make and sustain change remains central to improving cat-management outcomes. Containing cats is a particularly emotionally charged and value-laden issue, shaped by diverse caregiver beliefs about what constitutes a good life for their animals. These beliefs intersect with a range of practical and social factors that influence containment decisions, including cost, housing design, rental restrictions, and cultural norms that view roaming as natural or necessary for cats [16,17,18]. The presence of semi-owned cats, who receive some level of care but for whom no individual accepts full responsibility, further complicates accountability and limits the reach of containment initiatives [19,20,21]. Even among owners who express strong intentions to contain their cats, these intentions often fail to translate into consistent practice once emotional, social, or logistical challenges arise [22].

Behaviour-change theories offer valuable frameworks for analysing and influencing how individuals adopt new animal-care practices. The Transtheoretical Model (TTM) conceptualises behaviour change as a dynamic, staged process in which individuals move from *pre-contemplation* (limited awareness of a problem) to *contemplation* (acknowledging a problem but uncertain about change), *preparation* (gathering information and planning), *action* (implementing new behaviour), and *maintenance* (sustaining change over time) [23]. Complementing this model, the Capability–Opportunity–Motivation–Behaviour (COM-B) framework [24] emphasises that behaviour arises from the interaction of three core components: *capability* (knowledge and skills), *opportunity* (external factors that enable or constrain behaviour), and *motivation* (internal processes such as beliefs, values, and emotions).

Ma and McLeod [25] applied these concepts to cat containment in Australia. They identified six distinct owner profiles that differ in their beliefs, motivations, and perceived ability to act: *Freedom Defenders*, *Tolerant Guardians*, *Laissez-faire Landlords* (A and B subtypes), *Conscientious Caretakers*, and *Concerned Protectors*. The “movable middle” of this spectrum—primarily the *Laissez-faire Landlords*—represents the largest profile of owners. This profile had no strong opinions about containment, had no current intentions to change their cat containment behaviour and showed little motivation to contain their cats, whether framed from a cat welfare or a community frame. Behaviour-change interventions that use marketing to target this group’s motivation to contain their cats might therefore have the greatest impact on increasing their uptake of voluntary containment [26].

Because perspectives on cat containment differ widely across cat caregivers, effective communication requires identifying message frames that resonate with the target “moveable middle” without alienating cat caregivers from other audience segments [27]. For instance, messages centred solely on biodiversity protection may not resonate with cat caregivers who are motivated by their cat’s welfare [28,29]. At the same time, safety-based appeals may fail to connect with those concerned about ecological impacts. Motivational engagement offers a way to bridge these differences by appealing to shared emotions and identities, prompting owners to reflect on their values, increasing their willingness to seek information, and building their confidence. Given that mass-media campaigns have limited capacity to convey complex information within short attention spans [30], they are most effective when they first evoke emotional resonance and curiosity, motivating cat caregivers to actively pursue further guidance. Once motivation is activated, owners are more likely to acquire the knowledge and resources needed to sustain containment through self-directed learning and social reinforcement, thereby increasing their capability and, ideally, leading to gradual increases in containment behaviour.

Recognising the need for innovative approaches to motivating change with domestic cat management, the Royal Society for the Prevention of Cruelty to Animals (RSPCA) in New South Wales (NSW), Australia, implemented the *Keeping Cats Safe at Home (KCSAH)* project. Supported by an AUD 2.5 million grant from the NSW Government’s Environmental Trust, this four-year initiative (2021–2024) aimed to translate behavioural science into practice by promoting the voluntary containment of companion cats in a sustainable manner that considered feline welfare. Delivered across 11 local councils, the program sought to reduce both the ecological impacts and welfare risks associated with free-roaming cats through coordinated community engagement, education, and strategic partnerships. As part of this effort, the project team recognised the need for deeper insight into how cat owners interpret and respond to different message framings regarding roaming. In particular, it aimed to understand how behavioural, emotional, and motivational factors shape owner responses to containment campaigns and which types of messages are most likely to influence the “movable middle” of owners who remain undecided about change.

Accordingly, the present study used a focus-group approach to explore owner motivations and barriers to implementing cat containment and to assess owner responses to alternative behaviour-change campaign concepts. Specifically, the objectives were to: (1) develop a stronger understanding of the target audience’s perceptions of cat containment and roaming, and (2) analyse and interpret reactions to two distinct motivational campaign concepts designed to encourage voluntary containment.

## 2. Materials and Methods

### 2.1. Positionality Statement

Except for KLS, all the authors are current or former cat caregivers. We manage our own cats in different ways, and we have varying personal perspectives on cat containment. Owing to most of our backgrounds in the fields of animal welfare and veterinary science, we lean towards prioritising the welfare of the individual cat when considering how cats should be managed. We are all advocates for holistic cat management that balances the welfare of cats, their caregivers, the community, and wildlife.

### 2.2. Participant Recruitment

The study aimed to recruit approximately 20 cat owners to participate in the focus groups. The target population comprised individuals residing in New South Wales who owned at least one cat with outdoor access and who would be classified as *Laissez-faire Landlords* according to the six cat-owner profiles described by Ma and McLeod [25]. This group typically includes owners who are not highly engaged with cat-related issues, give limited consideration to how they manage their cats, and are not strongly motivated by concerns about their cats’ safety. They generally view allowing cats to roam as a balance between safety and behavioural expression. As such, these owners are considered to be in the pre-contemplation stage of behaviour change, characterised by neutral attitudes and limited reflection on their decision to allow their cats to roam. To reflect the demographic profile of Australian cat owners, we also sought to achieve a gender distribution of roughly 60–70% female and a mean age close to 46 years [1].

An invitation email containing information about the study and the link to a screening survey was distributed to 6148 subscribers of the RSPCA NSW email newsletter ‘*the Cat-ch Up*’ in February 2024. The survey included tailored screening questions (see Supplementary Material S1: Screening survey and items used for profile classification) and the Short Attachment to Pets Scale (SAPS) [31] to classify owners according to the profiles described by Ma and McLeod [25]. Additional invitations were sent to eligible participants from Ma and McLeod [25] who had previously consented to be recontacted for future studies.

A total of 37 individuals responded to the invitation. Of these, 22 (59%) met the eligibility criteria, and all 22 were subsequently contacted to participate. Based on responses and participant availability, 22 individuals participated in one of four focus groups, each comprising between four and six participants.

### 2.3. Concept Development

A contracted advertising agency developed two unique creative messaging concepts as part of a broader campaign to encourage cat containment. Each concept was produced in three formats to present to the focus groups: radio, video, and still imagery.

#### 2.3.1. Concept 1: The “Perfect Cat” Concept

**Figure 1:**
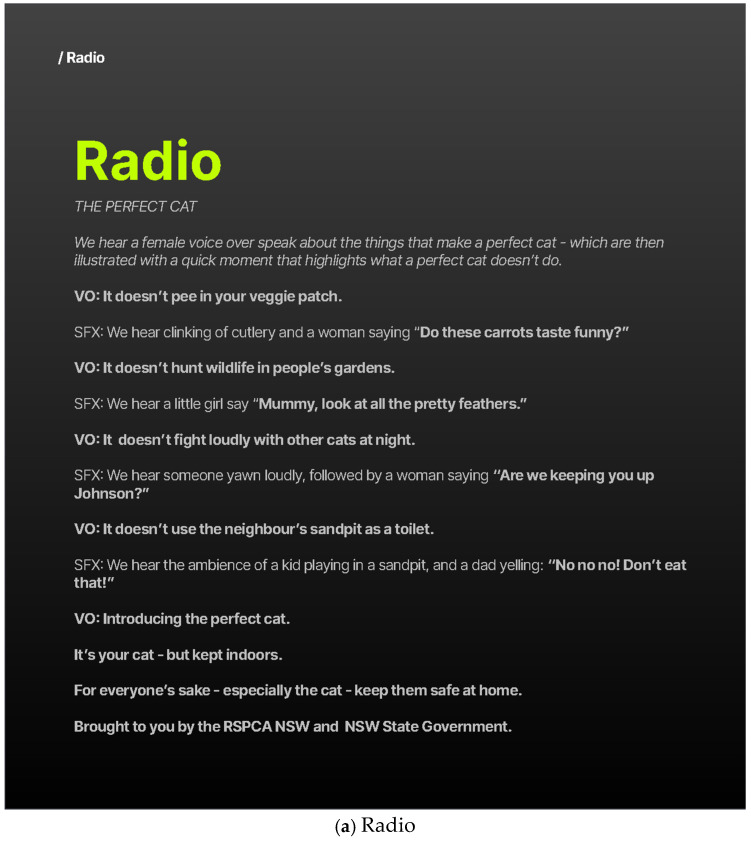

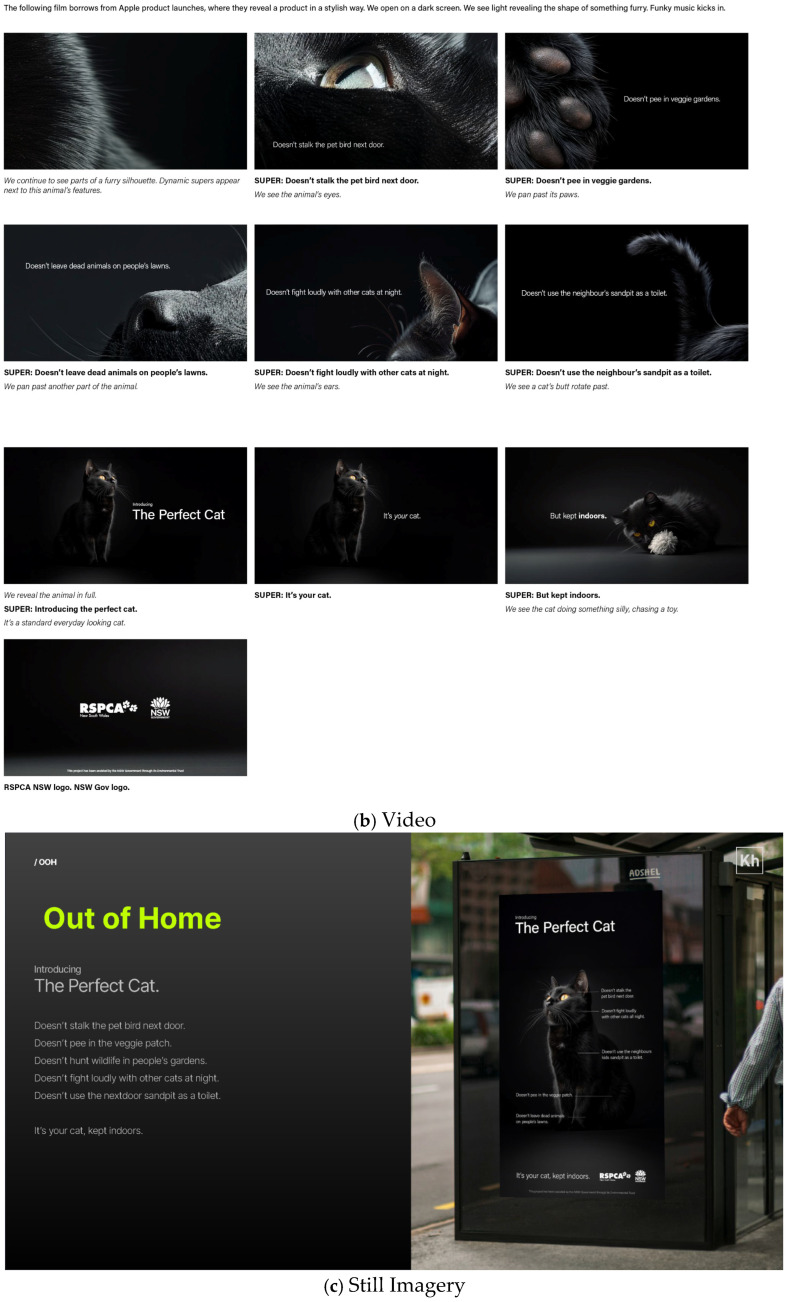
Illustrative examples of Concept 1—The Perfect Cat.

*Concept 1: The Perfect Cat* was developed around the insight that free-roaming cats can cause frustration for owners and neighbours through behaviours such as damaging gardens, disturbing wildlife, fighting, and creating nuisance noise (Figure 1). The creative intent was to reframe perceptions of cat ownership by introducing audiences to “the kind of cat Australia really needs”—a well-cared-for indoor cat. Using humour and relatable neighbourhood scenarios across multiple formats, the campaign contrasted the behaviours of roaming cats with those of a “perfect” indoor cat, concluding with the message that the perfect cat is “your cat—but kept indoors.”.

#### 2.3.2. Concept 2: The “Not All Cat Videos Are Funny” Concept

**Figure 2:**
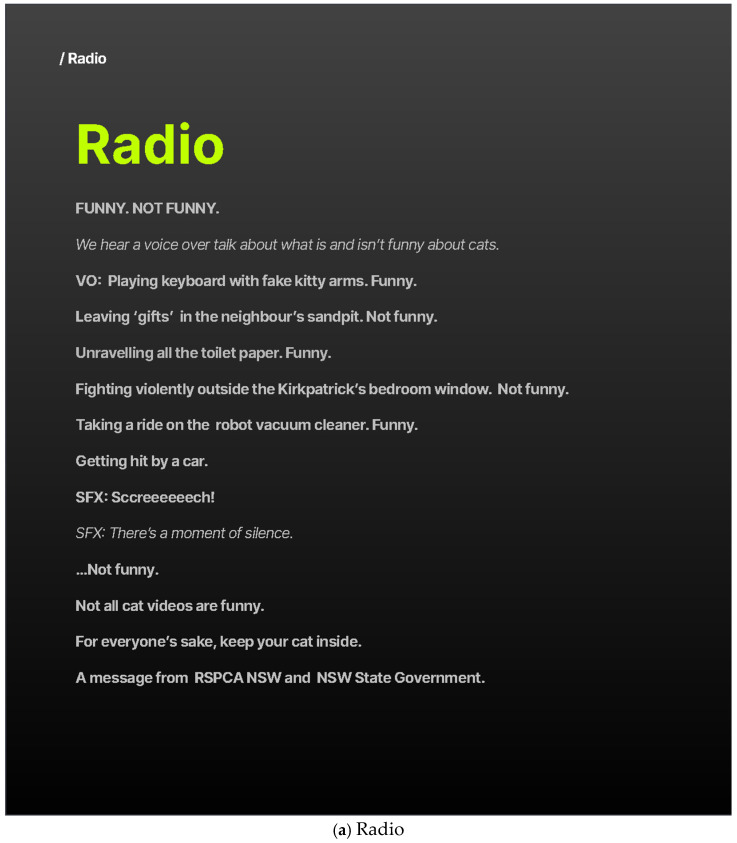

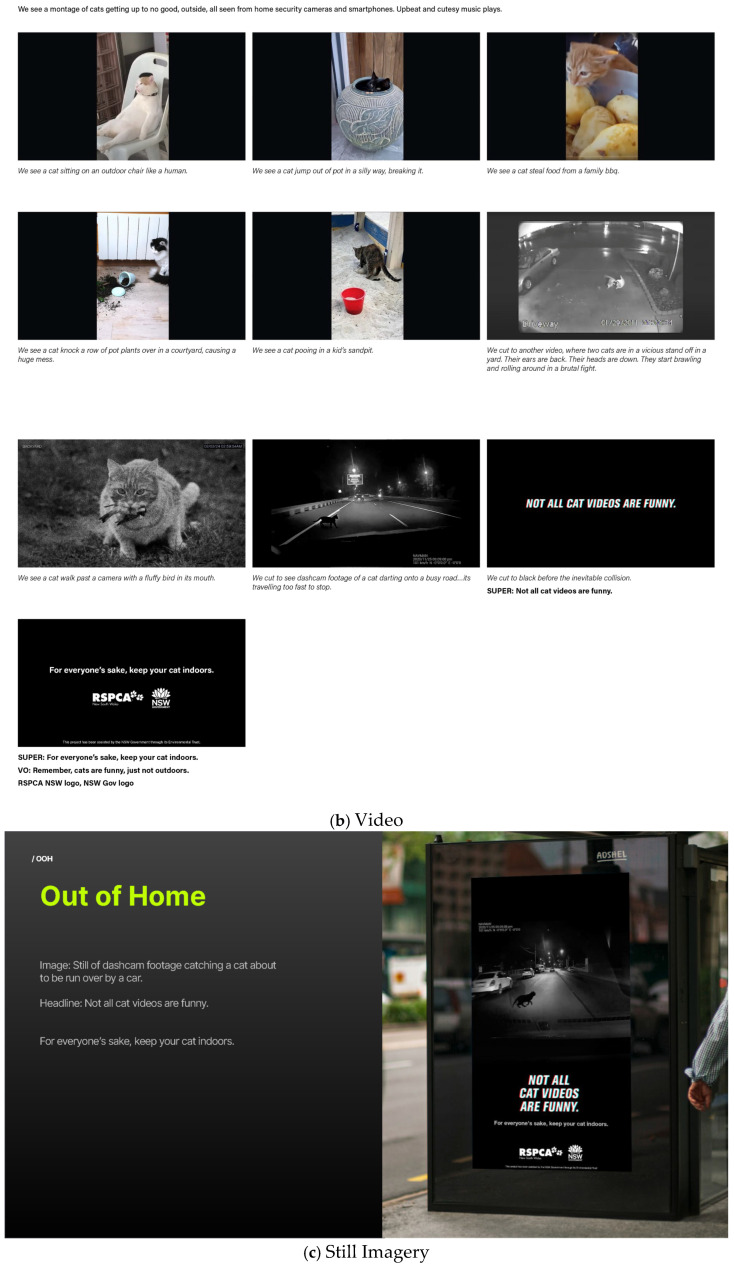
Illustrative examples of Concept 2—Not All Cat Videos are Funny.

*Concept 2: Not All Cat Videos Are Funny* was designed to juxtapose the light-hearted image of cats in popular media with the serious welfare and community issues arising from free-roaming behaviour (Figure 2). The concept was informed by the observation that, while cats are often celebrated for their amusing antics online, roaming behaviours can lead to adverse outcomes for both cats and communities. The creative approach employed a progressive “funny/not funny” narrative across radio, video, and still formats to shift audience perception from amusement to shock and concern. The storyline concluded with a sombre transition from humorous scenarios to a depiction of a cat being struck by a car, symbolising the real and preventable dangers faced by roaming cats. The message concluded with the statement, “Not all cat videos are funny,” reinforcing the campaign’s central call for caregivers to keep their cats safely contained.

### 2.4. Focus Group Facilitation

Focus groups were conducted online via Zoom in March 2024 and followed a structured discussion guide (see Supplementary Material S2: Focus group discussion guide) led by a trained facilitator. The guide included an introduction and icebreaker to encourage participation, followed by questions exploring participants’ relationships with their cats, cat containment behaviours and attitudes, and reactions to the two distinct advertisement concepts designed to encourage containment. The order of concept presentation was alternated between groups.

Participants received an information statement and consent form before the sessions, and verbal consent was confirmed before recording. Each focus group lasted approximately 90 min and was audio-recorded via Zoom.

### 2.5. Data Analysis

Audio recordings of the focus groups were transcribed verbatim and manually reviewed in Microsoft Word to ensure accuracy and readability. The research team used a reflexive thematic analysis approach [32], following an iterative process of familiarisation, coding, and theme development. We selected this approach because it is well-suited to identifying patterns across participant accounts in applied, contested topics. It maintains interpretive depth rather than treating analysis as a mere mechanical coding task. We employed Braun and Clarke’s reflexive thematic analysis, clearly framing themes as analytic results shaped by researcher engagement, rather than as categories that ‘emerge’ through consensus or inter-rater reliability, aligning with our positionality and the goal of guiding campaign design [33,34]. To ensure transparency and evaluability, we document our analytic process and results in accordance with current best practices and reporting standards for reflexive thematic analysis [35,36,37,38].

During familiarisation, the researchers read and re-read the transcripts, annotating ideas, reflections, and notable phrases, and recording initial observations in analytic memos. In the coding phase, salient text segments were highlighted and assigned short, descriptive, or conceptual codes that captured key ideas; multiple codes were applied where relevant. Reflexive notes were used to document analytic decisions and emerging insights throughout this process.

In the next stage, codes were clustered into preliminary themes based on conceptual similarity and relevance to the research questions. The team met regularly to review these clusters, identify central organising concepts, and map relationships between codes, subthemes, and overarching themes. During theme review, the coded extracts for each theme were re-examined to confirm internal coherence and to ensure that important perspectives were not overlooked.

Themes were then defined and named to capture their organising idea and analytic scope. Concise summaries were written for each theme, and labels were refined for clarity and resonance. All transcripts and codes were managed in NVivo (Version 14).

Finally, in the reporting phase, illustrative quotations were selected to exemplify each theme and are presented in Section 3 alongside analytical commentary. Ellipses indicate omitted words, and square brackets denote additions for clarity or context. Participant names were replaced with pseudonyms, and all cat names were standardised to “Biscuit” to preserve anonymity. Reflexive discussions within the research team throughout the analysis helped ensure transparency and consistency in interpretation while acknowledging the influence of the researcher’s standpoint.

### 2.6. Ethics Approval

Human ethics approval was granted by the University of Sydney Human Research Ethics Committee (Project Number 2024/165).

## 3. Results

In reflexive thematic analysis, themes are patterns of shared meaning organised around a central concept, developed through active interpretation rather than treated as a simple summary of discussion topics [33,34]. We therefore present each theme with an analytic narrative, supported by illustrative quotations, to make the interpretive basis of the findings transparent.

### 3.1. Participant Demographics

Of the 22 focus group participants, 14 (64%) identified as female and eight (36%) as male, with a mean age of 47 years. The majority (68%) were aged 40–54 years, with smaller proportions aged 25–39 years (19%), 55–60 years (9%), and over 60 years (5%). Most participants (64%; n = 14) owned a single cat, while 23% (n = 5) owned two, 9% (n = 2) owned three, and 5% (n = 1) owned four or more. All participants resided in New South Wales, including 20 in metropolitan areas and two in rural locations. In terms of housing, 73% (n = 16) lived in a house with a fenced outdoor space, 14% (n = 3) in an apartment or flat with limited outdoor access, 9% (n = 2) in a house with no fenced area, and 5% (n = 1) in a townhouse with a fenced courtyard. Eleven participants (50%) allowed their cat(s) to roam freely during the day but contained them at night, eight (36%) allowed unrestricted roaming, and three (14%) permitted only restricted outdoor access (e.g., within an escape-proof yard, on a lead, or under direct supervision).

### 3.2. Cat Containment Behaviours and Attitudes

**Impact of acquisition source and prior outdoor exposure on containment decisions**

Participants noted that how they acquired their cat strongly influenced their decision about containment. Those who obtained their cat as an adult, particularly through rehoming, adoption, or taking in a stray, were more reluctant to introduce containment. They felt that cats with prior outdoor experience had established habits or an inherent “right” to continue roaming, and that restricting access to the outdoors would compromise their cats’ welfare.

*“Our cat has owned us for four years, but we’ve only owned her for two … she slowly moved into our house. She was an outside cat which kind of coincides with the language that I’ve used in terms of her coming to us and adopting us, she remains that way with a bit of freedom.”*—Ethan

*“So, my Biscuit, she was originally an outdoor cat. I couldn’t keep her indoors because she’s very independent and she just wanted to be outside.”*—Harriet

Participants also reflected that cats raised indoors from kittenhood may be more content with containment because they have never experienced the outdoors. However, some participants still questioned whether such cats might instinctively long for natural environments.

*“People would tell me that it’s a bit cruel to keep them only indoors when I had an indoor cat. My own experience is you don’t miss what you don’t know … if they’re staying indoors, they never know the outdoors.”*—Abigail

*“If a cat doesn’t know any better and if it’s been brought up inside as a kitten then they probably don’t miss it, but I would still challenge that because indoor cats sit at the window and longingly look outside and I think any animal misses nature and being outside.”*—David

**Underestimation of risk and poor awareness of roaming behaviours**

Participants frequently justified allowing their cats to roam by citing anecdotal evidence that minimised perceived risks or impacts. Many asserted that their cats did not engage in behaviours associated with roaming risks or impacts such as hunting, fighting, or travelling far from home. They therefore posed little threat to wildlife or themselves. However, these claims were often followed by anecdotes describing cats exhibiting precisely those behaviours, suggesting a degree of cognitive dissonance or denial regarding the risks and impacts associated with free-roaming cats.

*“[One] day the cats brought in a live, and I stress that, live baby bandicoot. It was not bitten or hurt … [but] the next morning [it] perished … People say cats are vicious, but even last night we had another bandicoot outside and they don’t touch them, they know not to touch bandicoots.”*—Nancy

*“The furthest he’ll wander is pretty much to the end of the driveway. Maybe the next house down the road. That’s about it. He doesn’t really go any further.”*—Georgia

*“During the day I have never experienced her hunting. She brings the odd mouse which I guess is fine, but she’s never killed a possum or bird or anything … she brings us what she kills, so I kind of can tell what she’s doing. On the odd occasion, she brings a little lizard which makes me sad”*—David

**Overconfidence in feline cognition and capacity for self-regulation**

Closely related to these perceptions was a strong sense of caregiver confidence in their cats’ level of awareness and cognition, and ability to manage risk independently. Participants commonly described their cats as sensible, mature, or capable of avoiding harm, often attributing human-like judgment to their behaviour. This overconfidence appeared to reduce caregivers’ concern about potential dangers and led them to believe that containment was unnecessary.

*“There’s this children’s crossing right next to our house, so usually at night when my cat wants to get to the other side of the road, she loves to look both ways first and she checks and makes sure there are no cars coming either direction and then she just crosses the road with no problems at all.”*—Phil

*“Biscuit will catch a lizard like a little skink, but she knows she’s not allowed to. And if [our other cat] does anything that looks like it has an animal involved, he puts himself in the toilet to calm down.”*—Kim

*“Biscuit seems to have more maturity which sounds weird to say that about a cat but it felt like he was just that little bit more responsible.”*—Jerry

**Perceptions of cat personality shape containment decisions**

Caregivers frequently described their cats as having distinct personalities and interests that influenced their decisions about outdoor access. Many viewed roaming as an essential outlet for their cat’s curiosity, independence, and overall welfare, framing it as a matter of respecting the cat’s individual preferences rather than a management choice. Attempts to restrict outdoor access were often perceived as limiting the cat’s ability to exercise agency or to express certain behaviours.

*“Going outside was a non-negotiable. She needed that, and she requested it, and asked to go outside for her own mental health or for her own enjoyment. She enjoys basking in the sun or going to the toilet or jumping the fence and scratching trees and whatever, and I am not comfortable with denying that.”*—Ethan

*“I’d take him out on a leash because we lived on a very, very busy road and I just knew he would run out onto the road … it was the only way to let him get that curiosity out. Because if we kept him in, the longer we’d keep him he’d start becoming quite aggressive towards me … which is crazy to think of him now, he’s the most placid cat I’ve ever experienced.”*—Georgia

### 3.3. General Reactions to Cat Containment Campaigns

Participants shared several overarching responses to the campaign that were not specific to either creative concept.

**Denial of responsibility and minimisation of risk**

Many participants expressed discomfort with the overall message, distancing themselves from the behaviours depicted and questioning the campaign’s accuracy and relevance. Several denied that their cats engaged in such behaviours or perceived the risks presented as exaggerated.

*“I’ve never hit a cat and I drive every day. I think they’re making it seem like this is happening to everyone a lot … I don’t know many people that have actually ever hit a cat. I’m not saying it doesn’t happen, but I think they’re trying to make us think it happens more than it does.”*—Scott

Participants also redirected responsibility toward other perceived problems, such as dog attacks or environmental damage caused by people, suggesting that cats were unfairly targeted.

*“What about the dogs who are killing children, isn’t it more sensible to make an ad about how to keep your dog on a lead and how you train your dog properly to walk in the park. I’ve been attacked by dogs and bitten and I’ve been run over by dogs and hurt and yeah, maybe the odd cat kills something which is heartbreaking. It’s terrible, but I think there’s bigger fish to fry.”*—David

*“Nobody talks about keeping dogs inside … There are animals out there and there are people who don’t look after their other animals. So why are you targeting the cats that stay in the yard, that sit in their front yard, but don’t go roaming the streets? And I think it also lumps all animals into the same class as almost criminal, just because one cat does it, doesn’t mean all cats do that.”*—Kim

*“I find it funny bringing the issue of native animals to the forefront, I think the state government has destroyed a lot more habitats and wildlife through developments than we do daily”*—Nancy

Some felt the campaign implied moral judgment, prompting them to disengage. Those who already implemented partial containment measures (e.g., night curfews), who owned older cats who were used to roaming, or who lived regionally considered themselves outside the target audience.

*“My cats are already roaming and have been for 10 years, that won’t make a difference to me seeing that campaign. In fact, it would annoy me a little bit thinking about how that money could be spent because we know how much advertising costs.”*—Scott

*“I’m not the right audience because I don’t let my cat out at night for those reasons, but the message should be around. ‘Do you know, what you can do is, at night, bring the cat inside at night.”*—Claire

*“My cats are kept inside at night time, so that doesn’t relate to me.”*—Kim

*“If I lived in the middle of the city I probably wouldn’t allow my cat to roam, but I don’t have that issue here so I can’t speak on that.”*—Scott

**Preference for a solution-focused approach**

Participants felt the campaign identified problems but offered few practical solutions. Many expressed frustration at being told their management practices were wrong without being shown feasible alternatives.

*“I think the issue is that they don’t give solutions or at least suggestions, we all have busy lives and I guess most of us, we can’t be bothered about ‘Am I a good or bad cat owner and what can I do differently?’ Not many of us are going to take the time to go through this thought process.”*—Abigail

*“I would like to see something a little bit more practical, like how you’re going to keep your cat happy at home and what ways it can thrive, how that works in practice.”*—Zack

*“There’s no solutions or suggestions to how to help support cat owners who have cats where it’s a non-negotiable or … who might want to do this but really have trouble with keeping their cat inside and keeping the cat happy. So there’s no solution or support.”*—Ethan

Participants also recommended including clear information about risks to make the message more persuasive.

*“If you want me as a cat owner to listen, then really talk to me about the safety of my cat. Talk to me about ‘this many cats get run over every year… because they’re outdoors’, ‘this many cats get poisoned because of the fox poison that’s out there because they’re roaming’, talk to me about things that really matter for myself as a cat owner, meaning the safety of my cat.”*—Abigail

*“It depends on the percentage. They need to give us percentages and ratios, like ‘This is very high according to the population’. That’s what would be more reasonable.”*—Rachel

**Intention to avoid the campaign**

Some participants stated that their initial reaction was to disengage entirely, reporting that they would skip, mute, or turn off the advertisements.

*“I wouldn’t even get to the end of the ad … if somehow this was shown in a news article, because that’s the way it works, I would just close the clip and wouldn’t watch it … I don’t agree with [it] from a moral standpoint.”*—Nancy

*“I think the second [concept] was too fluffy for me. I don’t think in a long-term campaign I would watch that. I would probably just switch [it] off.”*—Isabel

*“I wouldn’t mute [concept 2]. After the first [concept] ad, I would mute and turn away or turn off every time it came on screen after.”*—Kim

**Limited perceived influence on behaviour**

Participants commented on the perceived efficacy of the campaign and the individual concepts, with several stating that it would not affect their behaviour.

*“You’d get me thinking about safety very generally, but I wouldn’t place too much weight on it … I would just say ‘how is this relevant to me?’ And I would say [it’s] to get me thinking about cat safety, but I wouldn’t make any drastic changes to what I’m already doing.”*—Fiona

*“It wouldn’t change my mind about how long I keep my cat in.”*—Oscar

*“Would anything in my behaviour change after watching that? No. Subconsciously? Probably no too.”*—Nancy

*“None of those ads would appeal to change my behaviour whatsoever to keep my cat inside, so I don’t feel they speak to me at all.”*—David

**Concerns about broader implications**

Several participants voiced concern that containment campaigns could fuel negative public attitudes toward cats or lead to future legislative requirements for mandatory containment.

*“I don’t know what they’re planning. I know that some councils are legislating cats being indoors. I think, I’m not too savvy on it, but I did have that thought as I was watching that ad, that maybe they’re heading towards increasing legislation.”*—Fiona

*“There’s already people who hate cats and are vigilantes about cats and this fuels the violence and hatred towards cats … which is quite terrifying.”*—Kim

*“The fact that at the end the RSPCA logo and the [NSW] government logos come up is like, you look at legislation. We’re not sort of asking you to do this, we’re not trying to convince you, we’re telling you eventually it’s going to [happen]. So that there was another sort of warning sign for me.”*—Victor

### 3.4. Reactions to Concept 1: “The Perfect Cat”

Participants generally found *Concept 1: The Perfect Cat* concept more palatable than *Concept 2: Not All Cat Videos are Funny*, though many did not feel personally represented by its message or imagery. While some appreciated the lighter tone, others viewed it as disconnected from their own experiences and relationships with their cats.

**“That isn’t my cat”**

Many participants felt the campaign did not accurately reflect their cat’s behaviour or personality. They described the featured cat as too specific or unrealistic and therefore struggled to connect with the message.

*“I don’t identify with this perfect cat because that’s not who my cat is.”*—Abigail

*“It’s not really showing a proper understanding that there are different types of cats.”*—Zack

*“So what came across to me mostly was ‘This is not my cat you’re talking about’. The cat that goes to the sand pit and the cat that has fights and keeps people up. So I didn’t relate to the advert at all because it wasn’t talking about my experience now… it didn’t engage me at all because all the things you said weren’t things that my cat does. So why would I listen to this advertising.”*—Taylor

**“I don’t want that cat”**

Some participants disliked the use of the term “perfect,” interpreting it as implying that their own cats were flawed. Many emphasised their affection for their cats, as they are, expressing no desire to change them to fit the campaign’s portrayal.

*“I feel like they’re trying to sell an iPhone or a Tesla or that sort of like high-end luxury product when sometimes our cats aren’t that, and we don’t want our cats to be that, we want them to be like people have mentioned, individuals. They’re not all the same. We don’t necessarily want a predictable product.”*—Ethan

*“The ‘Perfect’ is just sort of a little bit confronting for me anyway, because like others said, my cat’s not the perfect cat, it does all those things.”*—Fiona

**“This isn’t important”**

Several participants perceived the behaviours depicted in the concept—such as urinating in gardens or fighting—as minor issues affecting neighbours rather than matters of cat welfare. They suggested that focusing on the risks to cats themselves, such as road trauma, would have been more meaningful and motivating.

*“To me, we’re not talking about the cat owner really in this case, we’re talking about the neighbors of the cat owner… So the neighbors might listen to it and agree with it, but they’re not in a position to influence behaviour around cats.”*—Taylor

*“There was no mention of cats being run over which in my experience happens fairly often. And so you’re not appealing to the owner of the cat, really. Because that might be a reason why they might want to keep it in, so it doesn’t get run over.”*—Taylor

*“I would question, do the RSPCA really care about carrots tasting funny because a cat peed in a veggie garden, would they really care about that? … It’s clear that they care about cats getting out and killing birds or whatever wildlife, so just say that … You’re the RSPCA and that’s not really important. So it just seems like it’s a bit of saying something, but not really saying what they want to say. And therefore is not really going to be effective to the people that actually need to be convinced.”*—Claire

### 3.5. Reactions to Concept 2: “Not All Cat Videos Are Funny”

Participants reacted more strongly to *Concept 2: Not All Cat Videos Are Funny* than to *Concept 1: The Perfect Cat*. The highly emotive imagery and abrupt tonal shift between humour and tragedy elicited intense feelings of fear, guilt, shame and discomfort among viewers.

**“The concept is confronting”**

Participants frequently described the campaign as confronting or distressing. Several questioned the appropriateness of the imagery for public display, particularly in places frequented by children.

*“I didn’t like that still image of the cat and the car coming being something so visible at bus stops. It’s somewhere where kids are a lot and we can drive fear and shame for adults, but I don’t think that that’s something I want driven into my kid.”*—Jerry

*“The cat image on the poster at the bus shelter … the one with the cat with the bird in its mouth … that’s a little bit disturbing, but it’s also reality. And I think cat owners probably need to be aware of reality and it’s not going to change overnight, it’s a longer-term change.”*—Lottie

*“[It is] potentially traumatizing for certain people to see, people who have lost their cats to cars and things like that … I could see potentially people complaining about it in the same way that they complain about other sort of violence or demonstrations of death.”*—Victor

**“I feel scared and guilty”**

Participants recognised that the campaign deliberately used shock and fear to elicit emotional responses. While some viewed this as an effective way to prompt reflection, others found it manipulative, unpleasant, or counterproductive.

*“It’s just that horror side of it and it shows a deathly consequence rather than … a benefit and an advantage. It’s just this ‘Hey kids, if our cat goes outside, it’s gonna die now’. But that’s how it feels, very horrific.”*—Jerry

*“I suppose it’s showing that there’s a lovely side to cats but there’s also the devastating side and I think that the devastation on our natural or native wildlife is a good thing to draw attention to… [Concept 2] felt more meaningful to me but yeah, I understand what [the other participant is] saying about it is a bit offensive to some people I suppose, or a bit upsetting to some people, but you’ll never get that blanket sort of rule for everybody to keep everybody happy.”*—Lottie

*“I get that fear is a powerful emotion, and yes, it can drive a reaction or a behaviour, but is fear how you want to do it? It just feels too short, as I said you start feeling happy, then you feel guilty and then you’re meant to feel scared and it feels like it’s a lot of emotions they’re trying to make you feel in a 30 second ad … It does happen, but to me, it’s not necessarily going after the wrong emotions, but the way that it’s doing it doesn’t leave me with a good taste in my mouth.”*—Georgia

*“You kind of play on guilt and fear in those ads. I think that cats will get out, that’s what they do. That’s what outdoor creatures do and no matter how hard you try to keep them indoors, they will escape, and when they do people are going to be really scared that they’re going to get run over and stuff like that.”*—Harriet

Participants described how fear often translated into guilt and shame, leaving them feeling targeted and uncertain about how to respond.

*“It’s also the horrific-ness of the end of it, it sort of strikes that fear and horror and then makes me feel guilty because my cat loves being outside. So it just didn’t seem great with me. It made me uncomfortable to be honest.”*—Jerry

*“My first thought is that nobody changes their behaviour due to shame. There’s always a pushback if you shame someone, it doesn’t change behaviour. It makes them dig in … I think it adds shame and it adds burden to people who are already trying.”*—Kim

*“I just think to scare people into doing something is not a pleasant feeling. To inspire people to do something and to give them reasoning behind it rather than just ‘Your cat’s gonna get hit by a car and killed’. It just feels like an unpleasant way to get the message across. It’s a very valid point. I’m not denying that … but to come out with that, it’s a lot.”*—Georgia

## 4. Discussion

This study explored how cat owners within the “movable middle” segment, who allow roaming but are not strongly opposed to containment, manage their cats and respond to alternative campaign framings designed to encourage voluntary containment. Overall, the findings highlight that once owners have established routines and perceive their cats as enjoying or needing outdoor access, their motivation to change is low and emotional resistance to containment messages is high. Behavioural habits, reinforced by beliefs about feline expression of agency and welfare, appear to entrench roaming as the default norm for this group of cat caregivers, making later change substantially more difficult than influencing management practices early in cat ownership.

### 4.1. Influence of Acquisition Source

How caregivers acquired their cat played a central role in shaping their attitudes toward containment. Participants who had adopted adult cats, particularly those with outdoor access before adoption, were reluctant to introduce confinement, perceiving it as depriving their cat of something they already valued or enjoyed. National data indicate that around three in five cats in Australia are obtained through formal or semi-formal sources such as animal shelters (28%), breeders (14%), pet shops (11%), and veterinary clinics (5%) [1]. These settings, therefore, represent critical intervention points where caregivers can be encouraged and supported to establish containment routines early, before roaming becomes a habitual expectation for both owners and cats. Providing clear guidance, visual modelling, and incentives to create a home environment that allows cats to engage in highly motivated behaviours at the time of adoption or sale could help normalise containment as a routine part of cat ownership and improve feline welfare. Although such face-to-face channels have higher per-person costs and lower reach than mass digital campaigns, they may be more effective in achieving sustained behaviour change by fostering deeper engagement and reinforcing social norms over time [39]. In-person conversations with trusted advisors, such as veterinarians or shelter staff, provide valuable opportunities to address owner concerns directly, offer practical, context-specific solutions, and model positive containment practices. However, more robust data are needed on how effectively these frontline professionals currently engage in discussions about containment and feline welfare, and how their role could be strengthened within broader education and communication strategies.

### 4.2. Moral Framing of Established Freedoms

Some participants framed containment as a moral violation rather than a practical change. Caregivers of free-roaming cats often described outdoor access as a pre-existing right and felt a duty not to revoke it. This reasoning reflects a form of moral reasoning noted in previous research in which restricting access breaches a perceived rule of fairness or promise-keeping toward the cat [40,41]. Similar patterns have been identified in previous cat-management studies, where owners often prioritise their cat’s autonomy and overall welfare over external concerns such as wildlife protection [18,42]. This helps explain why net-outcome appeals (i.e., those that present statistics on biodiversity impacts, or cat welfare trade-offs) often fail.

A more workable route might be to inspire cat caregivers towards adopting an alternative duty towards their cats, such as ‘guardianship’ or ’harm-prevention’, so containment is understood as keeping faith with the cat rather than betraying them. Safety-framed messages are more persuasive for cat owners because they affirm the caregiver’s identity as a responsible guardian and position containment as an act of protection rather than deprivation [43,44]. In this framing, containment becomes an expression of duty of care rather than the withdrawal of a right. This approach aligns with broader health-communication evidence showing that behaviour change is more likely when messages avoid identity threat (or a perception that the person’s values or identity are being criticised) and instead build efficacy and frame the new behaviour as aligning with their values [45,46].

### 4.3. Perceptions of a Good Life

Cat caregivers’ interpretations of what constitutes a good life for their cats were central to their justification of roaming behaviours. Many described outdoor access as essential to their cat’s happiness, freedom, and overall welfare, equating time outside with opportunities for stimulation, exploration, and self-expression. This perception is consistent with previous research showing that owners frequently associate outdoor activity with natural behaviour, emotional fulfilment, and a good life for cats. At the same time, containment is often equated with cruelty and poorer welfare [28,47,48]. Participants in this study frequently expressed concern that full containment would lead to boredom, frustration, or behavioural problems, and several viewed roaming as necessary for maintaining their cat’s “independence” or “confidence.” This reasoning aligns with the *natural living orientation* to animal welfare described by Fraser et al. [49], which emphasises opportunities for animals to perform species-typical behaviours and live in environments consistent with what caregivers believed to be ‘feline nature.’ In this view, containment risks denying cats authenticity and the ability to exercise agency, whereas roaming affirms both. Campaigns grounded in safety (biological functioning orientation) or emotional comfort (affective state orientation) may therefore have appealed to orientations these owners did not prioritise. Recognising this alignment is critical: reframing containment as a way to *safely enable expression of agency and natural behaviour*—rather than restrict it—may better align with owners’ core welfare values [49,50].

### 4.4. Cognitive Dissonance with Cat Behaviour

Many participants expressed views that were internally inconsistent, simultaneously acknowledging potential dangers of roaming while downplaying their personal relevance. This pattern reflects cognitive dissonance, where conflicting beliefs about safety and freedom create psychological discomfort that is managed through rationalisation or selective attention [51]. Participants often insisted that their cats did not roam far, hunt, or cause nuisance, yet later described behaviours that contradicted these claims. Similar findings were reported by Crowley et al. [52,53], who observed that many cat owners regard hunting as a normal or even beneficial behaviour and feel limited personal responsibility to manage it. These interpretations enable owners to maintain a sense of themselves as caring and responsible while avoiding the moral tension between protecting their cat’s safety and minimising ecological harm. In this study, participants often reduced discomfort by externalising responsibility or diverting their concerns to unrelated issues, such as dog attacks or urban development. This process helps maintain a positive self-image and minimises perceived need for change, highlighting how emotional self-protection and selective reasoning can act as substantial barriers to adopting containment practices.

### 4.5. General Reactions to the Campaign

Overall, reactions to the campaign concepts were immediate and emotionally charged. Many participants dismissed elements of the messages as exaggerated or irrelevant, consistent with the defensive responses commonly observed when communications challenge existing routines or identities, particularly among individuals in the pre-contemplation stage of change [46,54]. A strong adverse emotional reaction does not necessarily indicate ineffectiveness; however, deliberate use of messaging likely to prompt negative emotional reactions, especially where these might cause psychological harm, warrants careful bioethical consideration. Research in emotional and fear-appeal processing shows that high-arousal content can stimulate reflection and motivate protective behaviour even when viewers report discomfort or claim they would avoid repeat exposure [55,56]. Participants nevertheless expressed a preference for messages that provided clear, practical solutions for keeping cats safe and content at home, rather than problem-focused or punitive appeals. This aligns with social marketing evidence that supportive, efficacy-enhancing content is critical for helping audiences translate initial emotional engagement into action [46,57].

Reactions to *Concept 1: The Perfect Cat* concept were generally mild and often ambivalent. Participants appreciated the lighter tone and professional production noted in the concept-testing report, but most did not identify with the cat portrayed or the scenarios depicted. The focus on neighbour nuisance (e.g., sandpits, vegetable gardens) felt detached from owners’ own motivations, which centred on their cat’s freedom to express its behaviour. The idea of a “perfect” cat was interpreted by many as prescriptive or judgmental rather than aspirational, alienating those who valued their cat’s individuality. Proof points such as “carrots tasting funny” were viewed as implausible or trivial, undermining message credibility. Participants wanted the campaign to recognise their affection for cats and to show realistic examples of how containment could enhance feline welfare.

The campaign’s references to “the perfect cat” and, potentially, to ideals of the “responsible cat owner” both draw on moralised language that can shape how audiences interpret containment messages. Each position’s moral worth differently; “responsible owner” focuses on human compliance with institutional norms, while “perfect cat” idealises particular feline traits or lifestyles, but both establish value hierarchies that imply moral judgment. Several participants rejected this framing, reading “perfect” as criticism of their cats’ individuality or of their own approach to care. From a relational ethics of care perspective, responsibility is best understood not as a fixed label but as an evolving practice embedded in relationships of attentiveness and responsiveness [58,59]. For many owners, caring well involves recognising and adapting to their cat’s individuality and ability to exercise agency rather than conforming to prescriptive ideals. Campaigns that appeal to this relational understanding of care—emphasising co-adaptation, trust, and protection—may foster reflection without triggering defensiveness or moral fatigue.

In contrast, *Concept 2: Not All Cat Videos Are Funny* elicited much stronger and more polarised responses. The juxtaposition of humour and shock achieved the intended attention grab, producing feelings of fear, guilt, and distress. Participants acknowledged the effectiveness of the final scene in conveying risk, and several noted that they would prefer not to watch it again. In motivational terms, however, this does not necessarily indicate disengagement. Research on emotional processing shows that high-arousal adverse content can trigger strong attention and memory encoding even when viewers avoid repeat exposure [55]. Fear-appeal models similarly demonstrate that negative affect can motivate protective behaviour when two conditions are met: the threat is perceived as personally relevant, and individuals feel capable of carrying out the recommended action [46]. Participant responses highlight the second condition, with many describing the message as compelling but wanting reassurance or clear guidance on what to do next. This aligns with evidence that, without efficacy cues, fear tends to elicit defensive avoidance rather than behaviour change [54].

From a social marketing perspective, an initial emotionally disruptive message can be strategically compelling when followed by simple, low-effort prompts that reinforce the desired behaviour [57,60]. In the context of cat containment, this means that a high-impact risk message may open the door to motivation. Still, subsequent materials must support capability and confidence by providing practical, achievable steps. These findings underscore the broader evidence on negative message framing: fear can activate motivation, but only when combined with efficacy, clear solution cues, and consistent reinforcement.

### 4.6. Limitations

This study was exploratory and based on a relatively small, self-selected sample of cat owners in New South Wales who had previously engaged with RSPCA communications or participated in related research. As a result, participants may have been more aware of cat management issues or more sympathetic to positive animal welfare messaging than the broader population of roaming cat owners. Conducting the focus groups online may also have favoured individuals who are comfortable discussing their views in a digital setting and who have stable internet access, potentially under-representing older, regional, or less digitally connected cat caregivers. The creative concepts were tested as stand-alone pieces rather than as part of an integrated campaign sequence, which limits the ability to predict real-world behavioural outcomes. Consequently, the findings should be interpreted as indicative of audience sentiment and motivational dynamics rather than as a direct measure of behavioural impact. Future studies could use quantitative methods with larger, more representative samples of cat caregivers to verify these qualitative findings.

### 4.7. Future Research Directions

Future studies should examine how motivational and capability-building elements can be more effectively integrated within containment campaigns. Controlled trials comparing different combinations of emotional framing, efficacy messaging, and practical support tools (for example, “first-week containment” checklists and environmental optimisation guidance) could clarify which sequences most effectively translate awareness into action. Research should also explore optimal timing and targeting, particularly interventions at key transition points such as cat adoption, relocation, or following a near-miss, even when owners may be more receptive to change.

In parallel, there is a need for stronger welfare and ethics-oriented evidence to guide both campaign design and public messaging. Future work could (1) develop and validate practical welfare assessment approaches for contained cats that are feasible for owners, veterinary teams, and researchers, and (2) examine how owners conceptualise feline welfare, including beliefs about agency, outdoor access, and what constitutes a good life for their cat. Mixed-methods studies linking owner beliefs and decision-making to observable behavioural indicators and overall welfare would help identify where support is most needed and what “good containment” looks like in practice. A focus is warranted on understanding the role of early life-experiences and socialisation in shaping a cat’s environmental preferences and behaviours as this might impact their ability to cope with conditions such as containment to an indoor environment. Likewise, further research could help to understanding how different cat personalities might be more or less suited to different types of homes. Ethical analyses should also consider how responsibility and acceptable trade-offs are framed across stakeholders, and how guidance can be designed to be transparent, proportionate, and responsive to uncertainty in the evidence base.

A broader, longitudinal evaluation combining attitudinal tracking with behavioural metrics (e.g., adoption-stage pledges, online resource use, or council-level compliance data) would help determine how communication strategies interact with policy, environmental design, and community supports to sustain containment behaviours over time.

## 5. Conclusions

Encouraging voluntary containment among established cat caregivers remains a complex behavioural, emotional, and moral challenge. Appeals that highlight risk, blame, or compliance with external norms are unlikely to succeed on their own, especially where roaming is normalized and where roaming is viewed as natural cat behavior and something that makes cats happy. The findings indicate that many owners reason from a deontological and natural living perspective, seeing outdoor access as a moral right and central to what they believe to be feline nature. Others approach care relationally, as an evolving practice of attentiveness and trust rather than adherence to prescriptive ideals.

Effective campaigns must therefore go beyond awareness-raising to connect with these moral and relational logics. Containment should be framed as an act of guardianship that keeps faith with the cat, protecting their ability to exercise agency and their safety, rather than as restriction or rule-following. At the same time, motivational appeals must be paired with clear, achievable steps that build capability and confidence, such as simple containment routines, indoor activity demonstrations, and information on cat care and how they communicate to ensure moves to containment also safeguard the cats’ welfare.

The most promising intervention point is likely to be at or soon after adoption, when new routines and expectations are being formed, and owners are most receptive to guidance. For the movable middle of cat owners, change is most likely when containment is presented as part of caring well in relationship with the cat.

## Data Availability

The raw data supporting the conclusions of this article will be made available by the authors on request.

## Supplementary Materials

The following supporting information can be downloaded at: https://www.mdpi.com/article/10.3390/ani16050784/s1. Supplementary Material S1: Screening survey for focus group participant recruitment; Supplementary Material S2: Focus group discussion guide.

## Abbreviations

The following abbreviations are used in this manuscript:

AUD	Australian Dollar
COM-B	Capability–Opportunity–Motivation–Behavior
KCSAH	Keeping Cats Safe at Home
NSW	New South Wales
RSPCA	Royal Society for the Prevention of Cruelty to Animals
SAPS	Short Attachment to Pets Scale
TTM	Transtheoretical Model
