# Supplementary material for: Understanding Australian Cat Caregiver Motivations and Reactions to Behaviour-Change Messaging on Cat Containment: Insights for Campaign Design
Source: Animals (Basel). 2026 Mar 3;16(5):784. doi: 10.3390/ani16050784 (PMC12985116; doi:10.3390/ani16050784)
Supplement: Supplementary file 1 [file animals-16-00784-s001.zip › animals-4173837-supplementary.pdf]

Supplementary Material S1: Screening survey for focus group participant recruitment.

To what extent do you agree or disagree with each of the following statements:

1. Cats should be free to roam wherever they choose.
2. Cats should be prevented from roaming freely to protect wildlife.
3. Cats should be prevented from roaming freely to keep them safe.
4. Cats should be prevented from roaming freely as it is good for their wellbeing.
5. Cats should be prevented from roaming freely as they can be viewed as a nuisance or unwelcome visitors by neighbours.
6. Cats do not like being prevented from roaming freely.
7. I am confident I can prevent my cat roaming freely at all times.
8. I am confident that I can provide everything my cat needs to ensure he/she is happy when not roaming.
9. The law should be changed to require pet cats to be kept at their owner's property at all times.
10. Preventing cats roaming freely is a practice that my family and friends would agree with.
11. Preventing cats roaming freely is a practice that my neighbours would agree with.
12. Preventing cats roaming freely is a practice that veterinarians would agree with.
13. Preventing cats roaming freely is a practice that other cat owners would agree with.
14. Cats should be prevented from roaming freely if in the future it is required by law.

Supplementary Material S2: Focus group discussion guide.

Part A: WELCOME AND INTRODUCTION (*10 MINUTES)*

Introduction

a. Introduce yourself as the moderator and explain the purpose of the focus group.

b. Briefly explain the objective of the research:

Exploration of creative concepts for a campaign that will encourage cat owners to keep their cats contained.

c. Emphasize the importance of participants' honest and open opinions and encourage them to share their thoughts and feelings.

Icebreaker

a. To set a comfortable atmosphere, get them to introduce themselves with their name and:

"Can you start by sharing a little about your experience as a cat owner?"

"How long have you had your cat(s), and what motivated you to become a cat owner?"

Part B: CAT CONTAINMENT BEHAVIOURS AND ATTITUDES (*30 MINUTES)*

Behaviours

- Can you share how much outdoor access your cat has and what influenced your decision to allow them both indoor and outdoor access?
- Can you describe a typical day for your cat, considering both indoor and outdoor activities? What kind of play, stimulation, or interaction does your cat experience in both environments?

Attitudes

- What are your thoughts about keeping your cats inside at night, and keeping your cats indoors 24/7?
- What concerns do you have about allowing your cat outdoors, and what benefits do you think they gain from outdoor exploration?
- What risks or dangers do you associate with letting your cat roam outdoors? Have you had any experiences or heard stories that influenced your views on this?
- What would be some of the benefits of keeping your cat indoors?

Intentions

- Would there be any situation that would make you consider keeping your cats indoors?

Part C: CONCEPTS TESTING (*40 MINUTES)*

We're now going to show you two concepts for an upcoming advertising campaign. These are not in a finished format yet, and your feedback in this focus group will help to finalise them. Please

be honest, and think about your initial thoughts and feelings when seeing each concept..

Concept 1

Please have a look at the following ad that, when finalised, will be in video format and could be shown on social media, Catch-up TV or YouTube, for example. This is in a draft format (i.e. not the finished video version), but this version will give you a good idea of what will be happening in the ad when it's finished.

[Show film Synopsis with storyboard]

Please now look at the following images.

[Show social media static / posters]

- Thinking about the video and the posters you've just seen, what messages do you think they convey?
- Still thinking about the video and the posters you've just seen, what, if anything, do you like about this advertising idea?
- Is there anything you don't like about this idea?
- And how do these ads make you feel?
- Would these ads make you think about keeping you cats indoors for a longer period than now?

Concept 2

Please have a look at the following ad that, when finalised, will be in video format and could be shown on social media, Catch-up TV or YouTube, for example. This is in a draft format (i.e. not the finished video version), but this version will give you a good idea of what will be happening in the ad when it's finished.

[Show film Synopsis with storyboard]

Please now look at the following images.

[Show social media static / posters]

- Thinking about the video and the posters you've just seen, what messages do you think they convey?
- Still thinking about the video and the posters you've just seen, what, if anything, do you like about this advertising idea?
- Is there anything you don't like about this idea?
- And how do these ads make you feel?
- Would these ads make you think about keeping you cats indoors for a longer period than now?

Concepts comparison

- If you had to choose one, which of these advertising concepts do you think will be most effective in getting other cat owners like you to think about keeping their cats indoors for longer periods of time?
- How do you think this campaign could be improved?

Part D: WRAP UP (*10 MINUTES*)

Inform participants that it is the end of the workshop and ask if they have any additional questions.

- Explain that incentives will be sent via email.
- Thank participants for their time and input.
